# Case report: A case of acute mastitis associated with reactive cutaneous capillary endothelial proliferation after camrelizumab treatment: A new immune-related adverse event

**DOI:** 10.3389/fimmu.2022.939873

**Published:** 2022-08-25

**Authors:** Ping-Shang Wu, Dan Xiong, Yang-Bo Feng, Luan Xiang, Jian Zhu

**Affiliations:** ^1^ Department of Thoracic Cardiovascular Surgery, The Third Hospital of Wuhan, Wuhan, China; ^2^ Department of Cadre Ward First, General Hospital of Central Theater Command of the People’s Liberation Army, Wuhan, China; ^3^ Department of Thoracic Surgery, Tangdu Hospital, Fourth Military Medical University, Xi’an, China; ^4^ Department of Thoracic Cardiovascular Surgery, General Hospital of Central Theater Command of the People’s Liberation Army, Wuhan, China

**Keywords:** mastitis, camrelizumab, programmed cell death protein 1, immunotherapy, lung adenocarcinoma

## Abstract

Lung cancer is a malignant tumor with the highest morbidity and mortality rate worldwide, and it seriously endangers human health. In recent years, immunotherapy has been widely used in lung cancer and has achieved great benefits, especially the application of promoting antitumor immune defense. However, immune-related adverse events (irAEs) caused by immune checkpoint inhibitors have received increasing attention, which largely limits their use. We report the first case of new acute mastitis caused by anti-PD1 inhibitors due to lung adenocarcinoma. A 65-year-old female patient came to our hospital for treatment with cough and shortness of breath for one month. Chest CT showed that the malignant tumor in the lower lobe of the right lung with pleural effusion had metastasized to many places, and then pleural effusion was taken for pathological examination. Pathological examination indicated that the pleural fluid originated from lung adenocarcinoma. Subsequently, the patient received platinum-containing dual-agent chemotherapy (carboplatin and pemetrexed disodium) combined with immunotherapy (camrelizumab). During treatment, the patient developed known adverse events and unreported acute mastitis. After stopping camrelizumab, the patient’s mastitis gradually improved. Our case shows that acute mastitis might be a new adverse event after the use of camrelizumab. Since this new adverse event has not been reported, we hope that oncology medical workers can obtain insight from our case and use it as a reference for the identification and management of irAEs.

## Introduction

Camrelizumab (SHR-1210) is a humanized monoclonal antibody against PD-1 (1). The effectiveness and safety of camrelizumab in a variety of tumors have been reported in a number of studies (2–5). Due to the resumption of T-cell surveillance, immune checkpoint inhibitors have completely changed the treatment of tumor characteristics of the immune system (6). Compared to chemotherapy or molecular targeted therapy, immune checkpoint blockade often produces a longer-lasting response. This may reflect the memory characteristics of the immune system. With the extensive use of immune checkpoint inhibitors in the clinic, irAEs caused by immune toxicity have begun to appear (7). irAEs are autoinflammatory to various organs and are caused by autoreactive T cells that act on normal tissues after being released (8). These irAEs are closely related to the role of the adaptive immune system and the evolution of responses to various internal threats (9). At present, there is no related report on the use of camrelizumab to cause acute mastitis in the literature. However, we identified a case of acute mastitis that was highly related to camrelizumab.

## Case report

**Figure 1:**
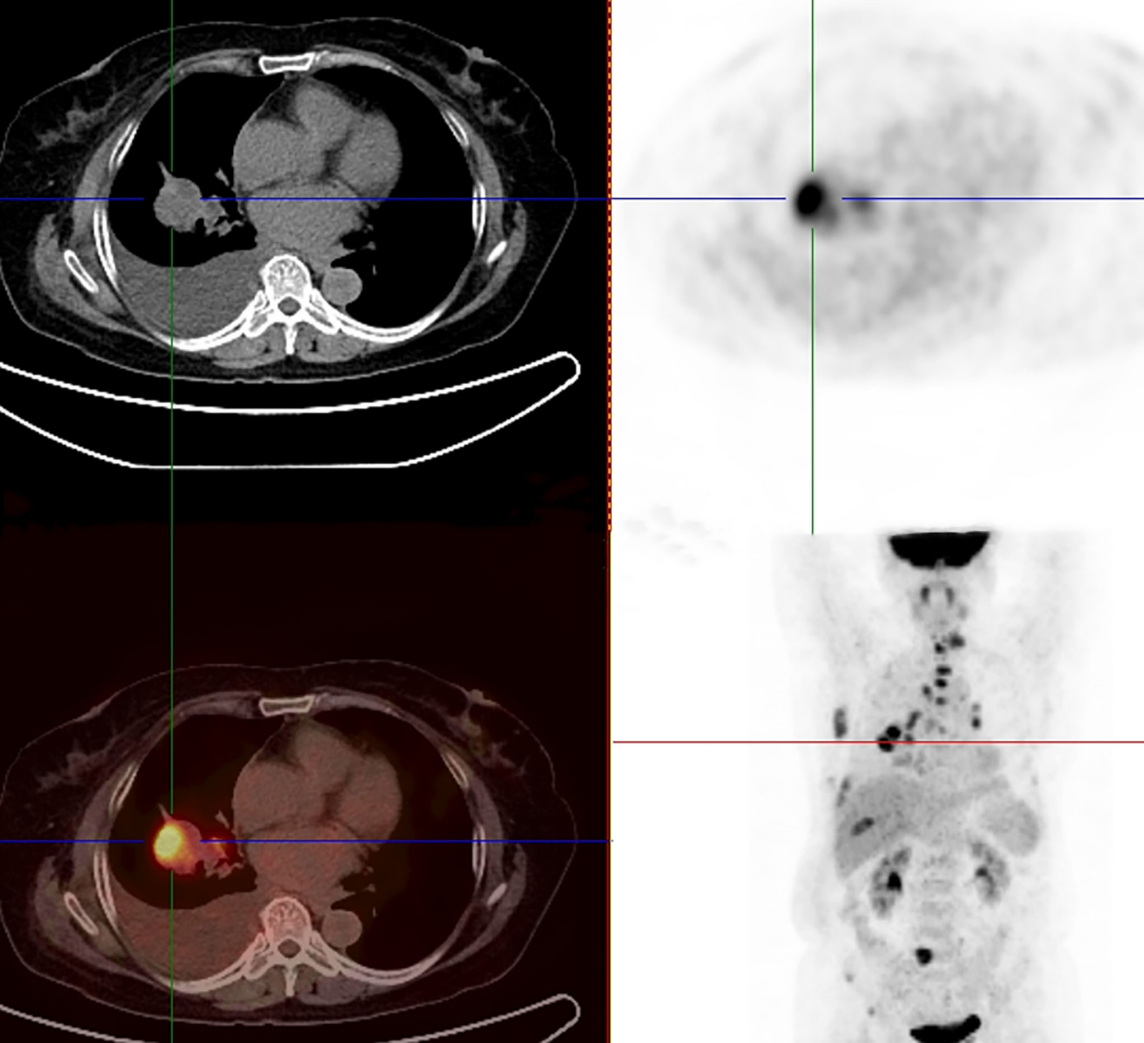
^18^F-FDG-PET/CT showed a right lower lobe malignant tumor (4.2 cm×3.3 cm×4.8 cm, SUVmax 9.6) with multiple right lung metastases and metastatic right pleural effusion.

**Figure 2:**
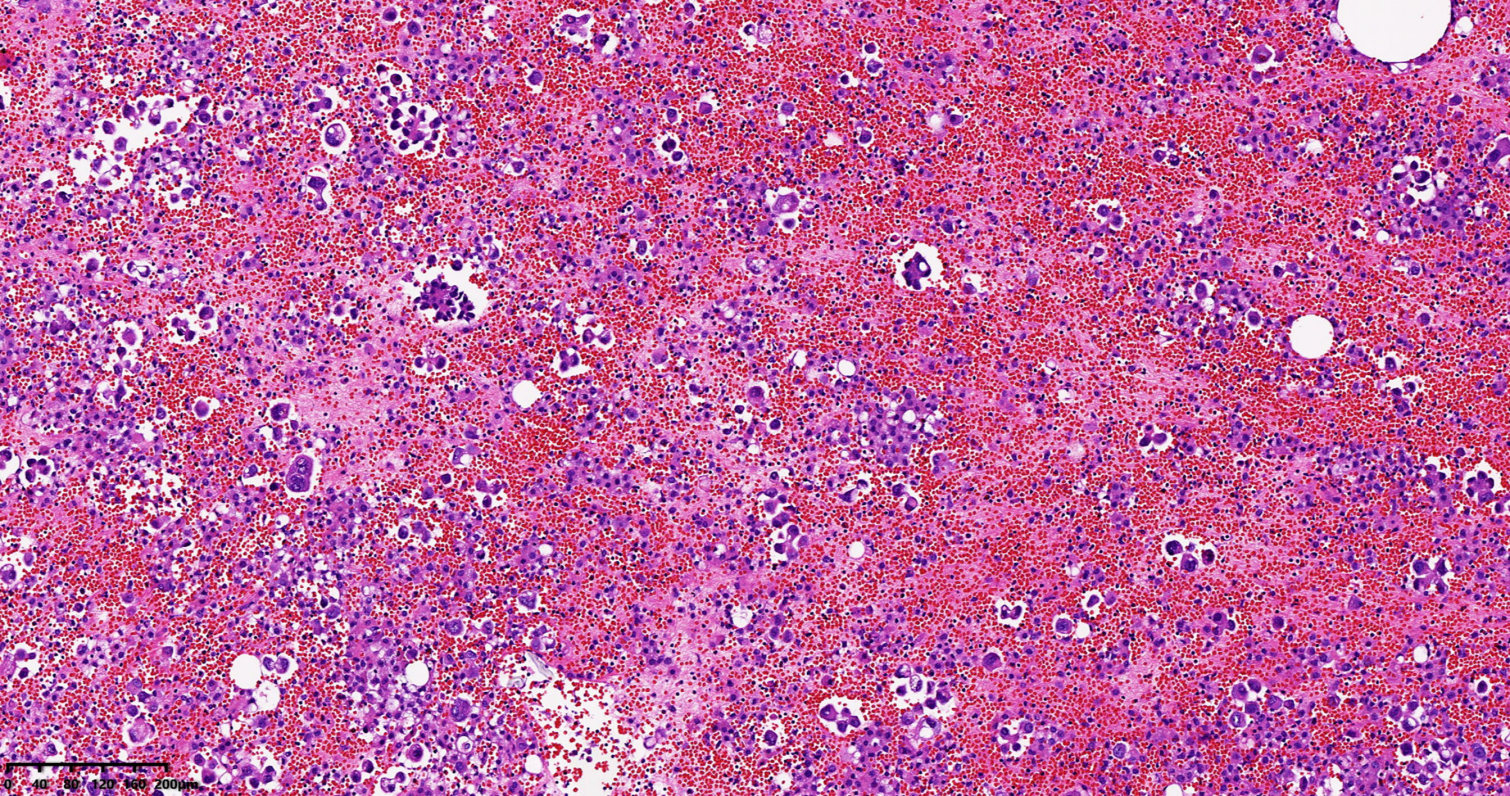
Pleural effusion sediment was confirmed to be adenocarcinoma. (hematoxylin-eosin stain, original magnification × 100).

**Figure 3:**
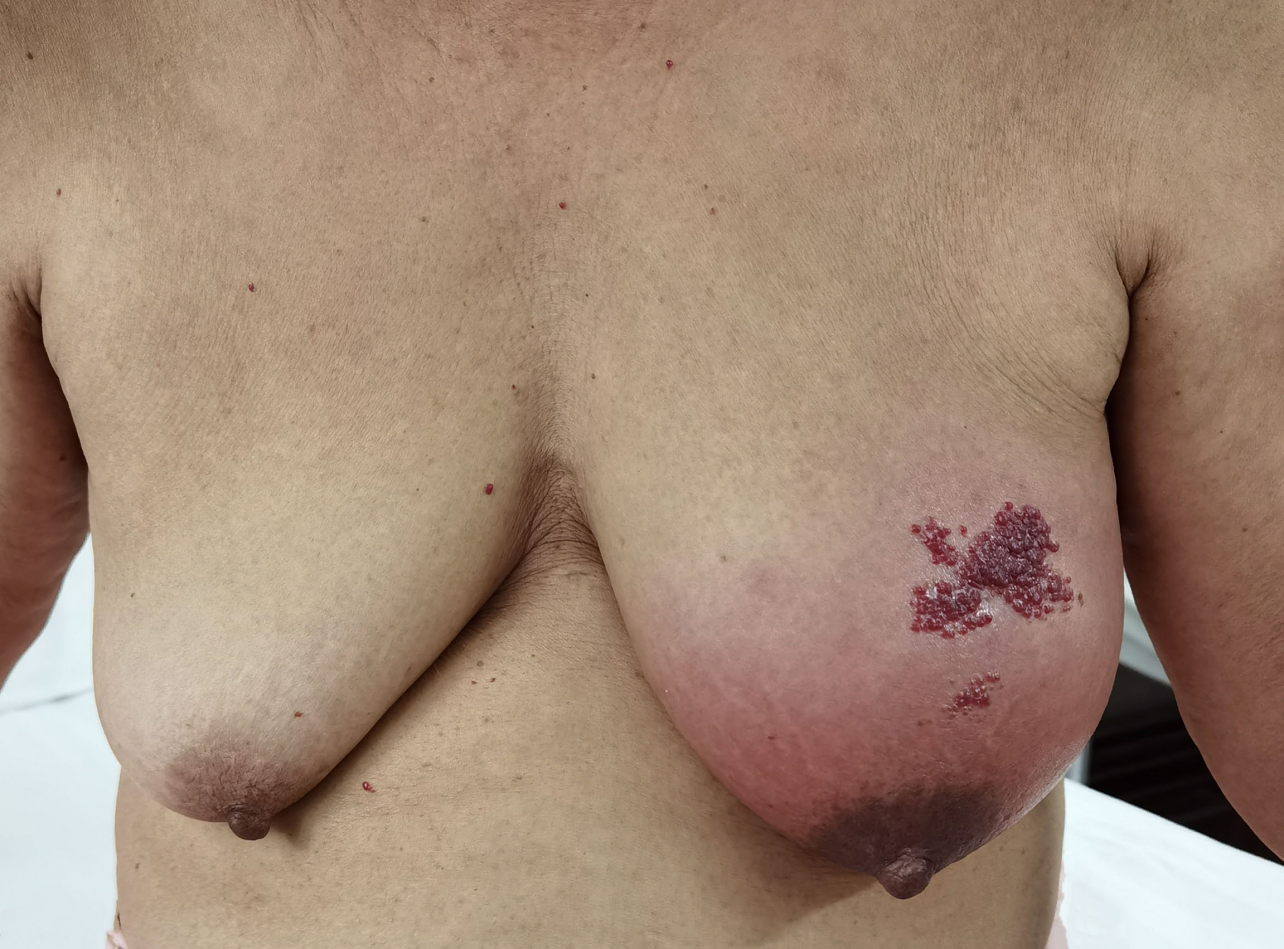
The appearance of inflammatory changes and “mulberry-like” reactive cutaneous capillary endothelial proliferation on the patient’s left breast skin.

**Figure 4:**
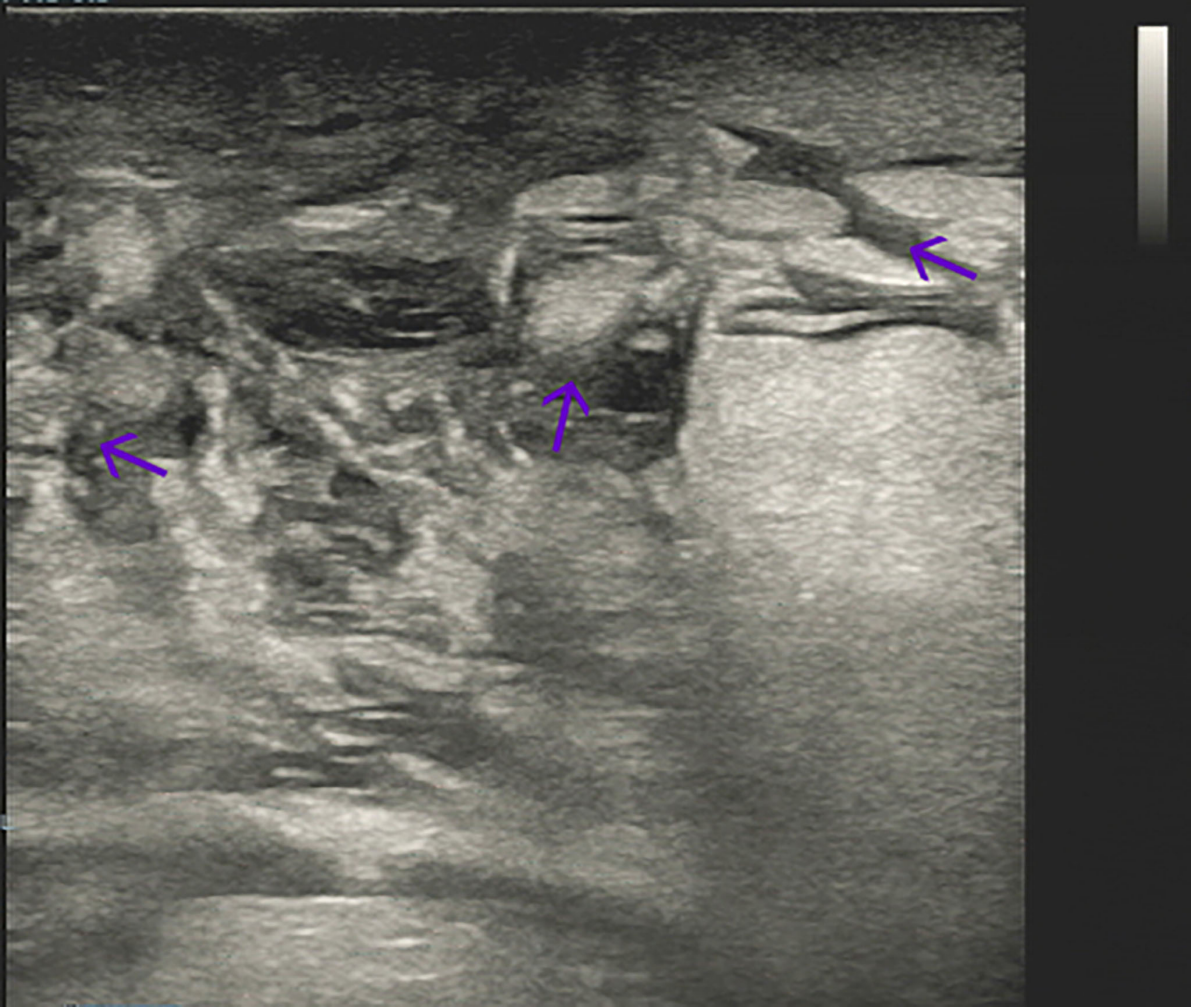
Breast ultrasound showed inflammatory changes in the left breast (the arrow points).

**Figure 5:**
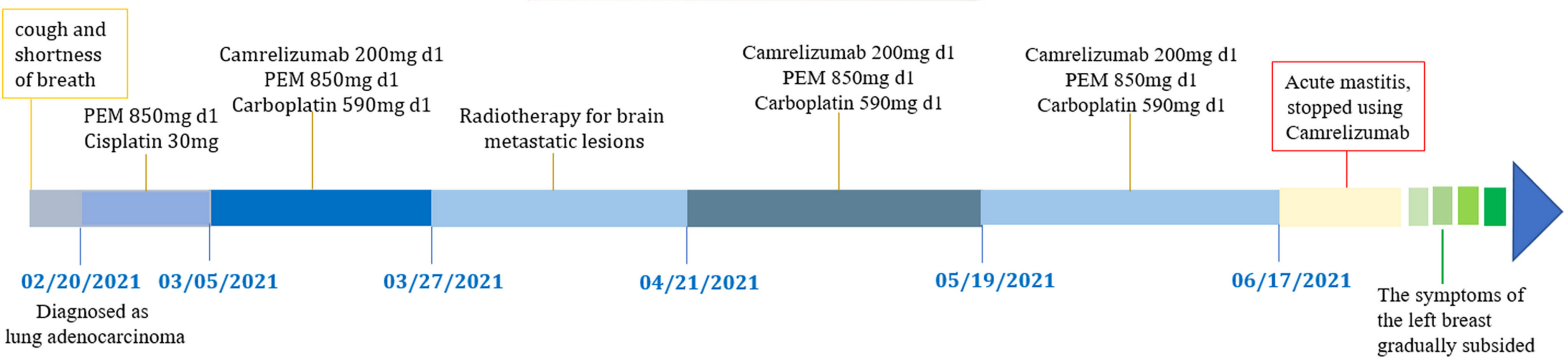
The timeline of different treatments for the patient’s entire treatment progression.

A 65-year-old female patient came to the hospital for coughing with shortness of breath for one month. She denied a history of smoking. Family members had no history of lung cancer or other genetic diseases. Physical examination showed that her breath sounds weakened on auscultation of her right lung and slightly thicker on auscultation of her left lung. Her heart rate was 82 beats per minute, the rate was the same, and no murmur was heard in the auscultation area of each valve. The abdomen was soft, and no tenderness, rebound pain, liver, spleen, or ribs were reached. No superficial enlarged lymph nodes were palpated. Chest CT showed a malignant tumor in the lower lobe of the right lung with pleural effusion that had metastasized to many places. Auxiliary examinations of whole-body PET/CT showed right lower lobe malignant tumor (4.2 cm×3.3 cm×4.8 cm, SUVmax 9.6, see **Figure 1**) with multiple right lung metastases, right pleural metastasis, right pleural effusion; longitudinal diaphragm and bilateral hilar lymph node metastasis; multiple bone metastases all over the body, pathological fracture of the 10th posterior rib on the right side; low-density shadow of the left frontal lobe on the head, no abnormal radioactive uptake. Head-enhanced magnetic resonance imaging showed multiple foci with abnormal enhancement in the brain, which were considered metastases. Tumor markers showed serum CEA 30.65 ng/ml and NSE 13.48 ng/ml. Pleural fluid CEA>1000.00 ng/ml. Pleural fluid immunohistochemistry showed TTF1 positivity, CK7 positivity, Napsin A positivity, CK5/6 (mesothelial cell positivity), P40 negativity, MOC31 positivity, CR (mesothelial cell positivity), PCK positivity, EMA positivity, and LCA (lymphocyte positivity). Pleural effusion sediment was embedded, and cancer cells could be seen under the microscope. Combined with clinical history and immunohistochemistry, the source was lung adenocarcinoma (see **Figure 2**). Common mutation gene detection received a negative result. She was diagnosed with lung adenocarcinoma (T_4_N_3_M_1c_) and was given 1 chemotherapy (pemetrexed disodium 880 mg d1) and 1 chest cavity infusion chemotherapy (cisplatin). Because the treatment was not effective, the treatment plan was subsequently modified. She received chemotherapy combined with immunotherapy (PEM 500 mg/m2 d1, CBP AUC=5 d1, Q3 W; camrelizumab 200 mg d1). In consideration of the patient’s metastatic brain lesions, local radiotherapy to the brain was decided for the next course (before the second using camrelizumab) of treatment after Multi-Disciplinary Treatment (MDT) discussion. The treatment went well, and the tumor was effectively controlled. Then, the patient received the same dose of chemotherapy combined with immunotherapy for a second time. After the second use of immunotherapy (camrelizumab), reactive cutaneous capillary endothelial proliferation (RCCEP) appeared on the patient’s left breast skin and was “mulberry-like”. Then, RCCEP was significantly aggravated by the third use of camrelizumab. Moreover, the patient felt swelling, pain and local skin high temperature of the left breast. Physical examination revealed enlargement of the left breast with pain, swelling, erythema, and warmth (see **Figure 3**). The right breast, including skin, was normal. The ultrasound showed inflammatory changes with typical “finger-like” tubular formations emerging from hypoechoic areas in the left breast, no obvious effusion, and a poor blood flow signal (see **Figure 4** where the arrow points). Considering that the patient’s left breast had no obvious pus accumulation and no purulent secretions, the white blood cell count, erythrocyte sedimentation rate and other inflammatory indicators were not abnormal, and she was not recommended for surgery or anti-infection treatment. However, further use of camrelizumab was not recommended. Then, the symptoms of pain, swelling, erythema, and warmth of the breast gradually subsided according to the patient’s feedback after giving up the fourth session of camrelizumab (the timeline of different treatments for the patient’s entire treatment progression see **Figure 5**). According to Naranjo’s adverse drug reaction score sheet (see **Table 1**), acute mastitis is most likely caused by camrelizumab.

**Table 1:** Naranjo’s assessment scale in the evaluation of adverse drug reactions.

Related issues	results	score
1. Is there any previous conclusive report on this ADR?	no	0
2. Does the ADR occur after the use of suspicious drugs?	yes	2
3. Does the ADR get remission after drug withdrawal or anti-drug application?	yes	1
4. Is the ADR repeated after the use of the suspected drug again? 5. Is there any other reason that can cause the ADR alone? 6. Does the ADR recur after placebo? 7. Does the drug reach toxic concentration in blood or other body fluids?	unknown no unknown unknown	0 2 0 0
8. Does the ADR aggravate with the increase of dose or alleviate with the decrease of dose? 9. Has the patient ever been exposed to the same or similar drugs and had similar reactions? 10. Is there any objective evidence to confirm the reaction?	unknown no no	0 0 0
Total score		5

Naranjo’s score ≥ 9 points: definite, 5-8 points: probable, 1-4 points: possible, ≤ 0 points: doubtful.

## Discussion

Lung cancer is a malignant tumor with the highest morbidity and mortality rates worldwide and is an important disease that must be addressed to maintain human health. Lung cancer can be divided into small cell lung cancer and non-small cell lung cancer, the latter accounting for approximately 85% (10–12). With the approval of a variety of immune checkpoint inhibitors for the treatment of lung cancer, the treatment of lung cancer has entered a new era (13, 14).

Immunotherapy has become a popular and promising treatment method to fight tumors by enhancing the function of the patient’s immune system. Immune checkpoint inhibitors are an important means of immunotherapy, and their efficacy in a variety of tumors has been widely confirmed (15). Immune checkpoint inhibitors enhance antitumor immunity by blocking T-cell cosuppressive molecules (negative regulators). In contrast, excessive immune responses after the immune checkpoint are blocked can damage normal tissue, thus causing irAEs. At present, many irAEs are autoinflammatory rather than autoimmune (16). For example, a cytokine release syndrome caused by immune checkpoint inhibitors. irAEs may occur in any organ (16). However, irAEs mainly occur in endocrine glands, skin, liver, etc. (17). irAEs often occur within a few weeks to a few months after the start of treatment. However, it can happen at any time, even after stopping the drug. Usually, adverse events of skin diseases appear first. When irAEs occur, common treatment methods include delaying the use of immune checkpoint inhibitors and using glucocorticoids or immunosuppressive agents to induce temporary immune suppression (18). The cause of irAEs may be related to genetic factors and the microbial composition of the patient’s intestinal flora (19–21). The severity of irAEs does not imply the pros and cons of immune checkpoint inhibitors in their anticancer effects (15). The possible mechanisms of irAEs include enhanced activity of T cells against antigens in tumors and normal tissues, increased levels of original autoantibodies, and increased levels of inflammatory cytokines (15).

Camrelizumab is an anti-PD1 humanized IgG4-kappa monoclonal antibody that exerts an anticancer effect by blocking the binding of PD-1 and PD-L1 and activating T cells to kill tumor cells (22). It was first approved for the treatment of patients with Hodgkin’s lymphoma in China in September 2015. It was approved for the treatment of lung cancer on June 19, 2020. Studies have demonstrated that camrelizumab exhibits a high affinity for PD1 and a high occupancy rate of circulating T lymphocyte receptors (22). Moreover, the binding epitope of camrelizumab is different from those of nivolumab and pembrolizumab. The irAEs faced by patients receiving camrelizumab treatment mainly include reactive cutaneous capillary endothelial proliferation (RCCEP), anemia, fever, fatigue, hypothyroidism, etc. (22, 23). Unlike other PD1 inhibitors, RCCEPs appear to be unique to patients taking camrelizumab. Apparently, this female patient also developed RCCEPs in the skin of the left breast after using camrelizumab. When the breast symptoms (swelling, pain, and local skin high temperature of the left breast) started, we thought it was RCCEPs. However, MDT discussion experts disagreed with this diagnosis. The reasons are as follows: the breast has obvious inflammation symptoms of redness, heat, and pain. Second, the volume of the affected breast was significantly larger than that of the contralateral breast. Third, the area of breast redness is almost the entire left breast, while RCCEPs are limited to part of the upper quadrant of the left breast. Fourth, breast ultrasound found that the left breast lesions were within the glands, not the skin layer of the breast.

Mastitis is an inflammatory condition of the breast with common symptoms of pain, swelling, erythema, warmth, and fever (24). Mastitis includes two kinds, namely, puerperal mastitis and nonpuerperal mastitis (25, 26). As with other mastitis, all types of mastitis are more common in one side, may also occur simultaneously in both sides (25–28). Nonpuerperal mastitis contains all causes of changes in breast and nipple inflammation that are not related to breastfeeding (29). It often lacks the typical local manifestations of redness, swelling, heat, pain, and systemic symptoms such as fever, chills, and fatigue. The pathological types include mammary duct ectasia/periductal mastitis and granulomatous lobular mastitis. The above mastitis often has abscess formation, but there is no abscess formation in this case of acute mastitis. We speculate that this type of mastitis does not seem to be different from nonlactating mastitis. This mastitis seems to be a new type of immune-related acute mastitis that occurs after RCCEPs caused using camrelizumab.

We reviewed the patient’s treatment history. Obviously, chemotherapy drugs will not cause mastitis. Previous studies showed that radiotherapy could influence mastitis although irradiated skin (29). However, our case had been treated with local radiotherapy to the brain, which did not involve the breasts or chest. That is, the mastitis in this case was not radioactive mastitis. Camrelizumab was the only suspected drug added before the onset of the condition. After we stopped camrelizumab, the acute mastitis improved. Next, we continued to follow up for 3 months after stopping the drug, and there were no more seizures. The patient understood the inferred diagnosis and was satisfied with the treatment result. Therefore, we highly suspect that the use of camrelizumab causes these new unreported irAEs (acute mastitis). Of course, we still need more sufficient evidence to confirm this finding. These tasks need to be further improved in future research. We hope that our medical record report will strengthen clinicians’ understanding of irAEs and improve their management capabilities for irAEs. At the same time, we hope to promote research on irAE mechanisms to speed up the optimization and improvement of immunotherapy drugs.

There are limitations to this case report. First, no histopathological evidence was obtained for this patient. Therefore, the diagnosis is based only on the patient’s symptoms and ultrasound examination. Second, we suspect that the use of camrelizumab causes new unreported acute mastitis based on this case report. More evidence needs to be confirmed locally with a large multicenter sample. However, that does not detract from the revelation with this report.

## Conclusion

Immunotherapy has greatly changed the treatment pattern of lung cancer and brought significant benefits to patients. However, when choosing immunotherapy, we should determine the indications and pay attention to the irAEs that may result in time. Our case showed that camrelizumab may cause one of the new irAEs, acute mastitis. This provides clues and a basis for the identification and management of irAEs.

## Data availability statement

The original contributions presented in the study are included in the article/supplementary material. Further inquiries can be directed to the corresponding authors.

## Ethics statement

Written informed consent was obtained from the individual(s) for the publication of any potentially identifiable images or data included in this article.

## Author contributions

P-SW and Y-BF offered the case and collected the data. DX and JZ prepared the manuscript. JZ and LX revised the manuscript. All authors contributed to the article and approved the submitted version.

## Funding

This report was supported by grants from Yuying Plan with Growth Project of General Hospital of Central Theater Command of the People’s Liberation Army, CHINA [No. ZZYCZ202106].

## Conflict of interest

The authors declare that the research was conducted in the absence of any commercial or financial relationships that could be construed as a potential conflict of interest.

## Publisher’s note

All claims expressed in this article are solely those of the authors and do not necessarily represent those of their affiliated organizations, or those of the publisher, the editors and the reviewers. Any product that may be evaluated in this article, or claim that may be made by its manufacturer, is not guaranteed or endorsed by the publisher.
